# Supplementary material for: A coordinated progression of progenitor cell states initiates urinary tract development
Source: Nat Commun. 2021 May 11;12:2627. doi: 10.1038/s41467-021-22931-5 (PMC8113267; doi:10.1038/s41467-021-22931-5)
Supplement: Supplementary file 1 — Supplementary Information [file 41467_2021_22931_MOESM1_ESM.pdf]

Supplementary information

A coordinated progression of progenitor cell states initiates urinary tract development

Sanchez-Ferras et al.

Supplementary Fig.1

a

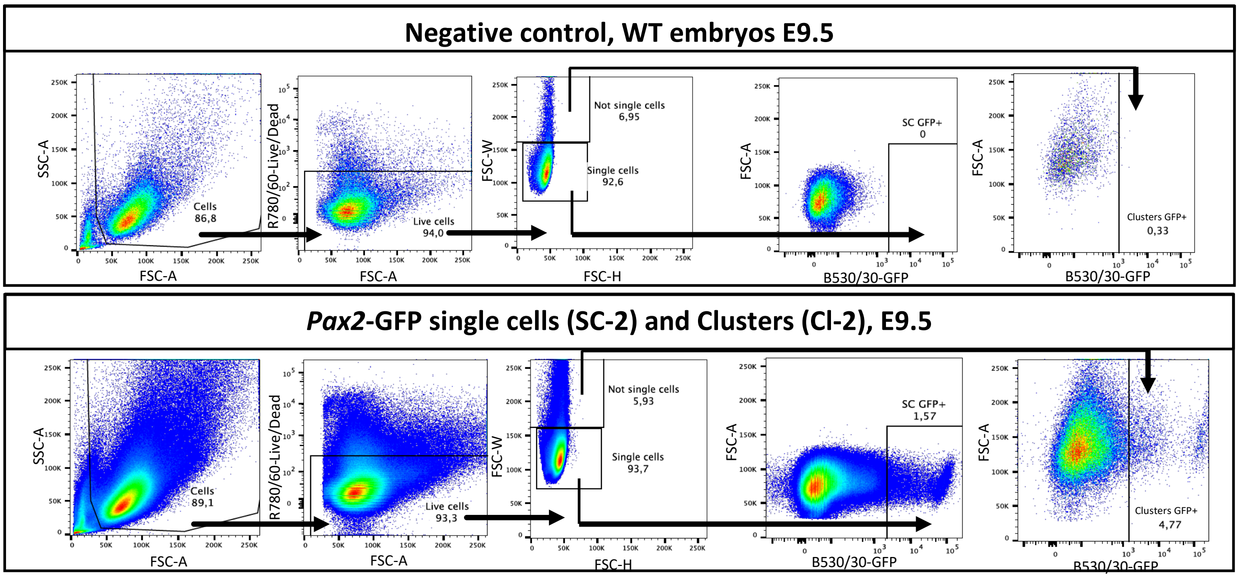

b

Library	# embryos	Embryonic day (Somite stage)	# Cell/Cluster sorted	cDNA yield (ng)	Sequence Ready Yield (ng)	# Cell/Cluster/ spot counts	# Reads per Cell/Cluster/spot
Pax2-GFP SC-2	3	E9.5 (21)	13417	1251	415	3415	47254
Pax2-GFP Cl-2	3	E9.5 (21)	2658	594	667	1346	106787
Pax2-GFP SC-3	3	E9.5 (18)	12178	1062	294	2642	57715
Pax2-GFP; Gata3 KO SC-3	3	E9.5 (18)	11774	1234	452	3666	46846
Pax2-GFP SC-4	4	E8.75 (11)	43147	1255	1657	6860	34418
Pax2-GFP SC-5	3	E9.0 (14)	15760	563	1027	2602	95282
(50% Epcam+) SC-6	4	E11.5	19895	1544	514	3549	44035
Visium-A1	3	E9.5 (21)	-	182	74	198	499766
Visium-B1	3	E9.5 (21)	-	161	92	222	466413
Visium-C1	3	E9.5 (21)	-	141	71	192	628606
Visium-D1	3	E9.5 (21)	-	139	69	178	657266

c **d**

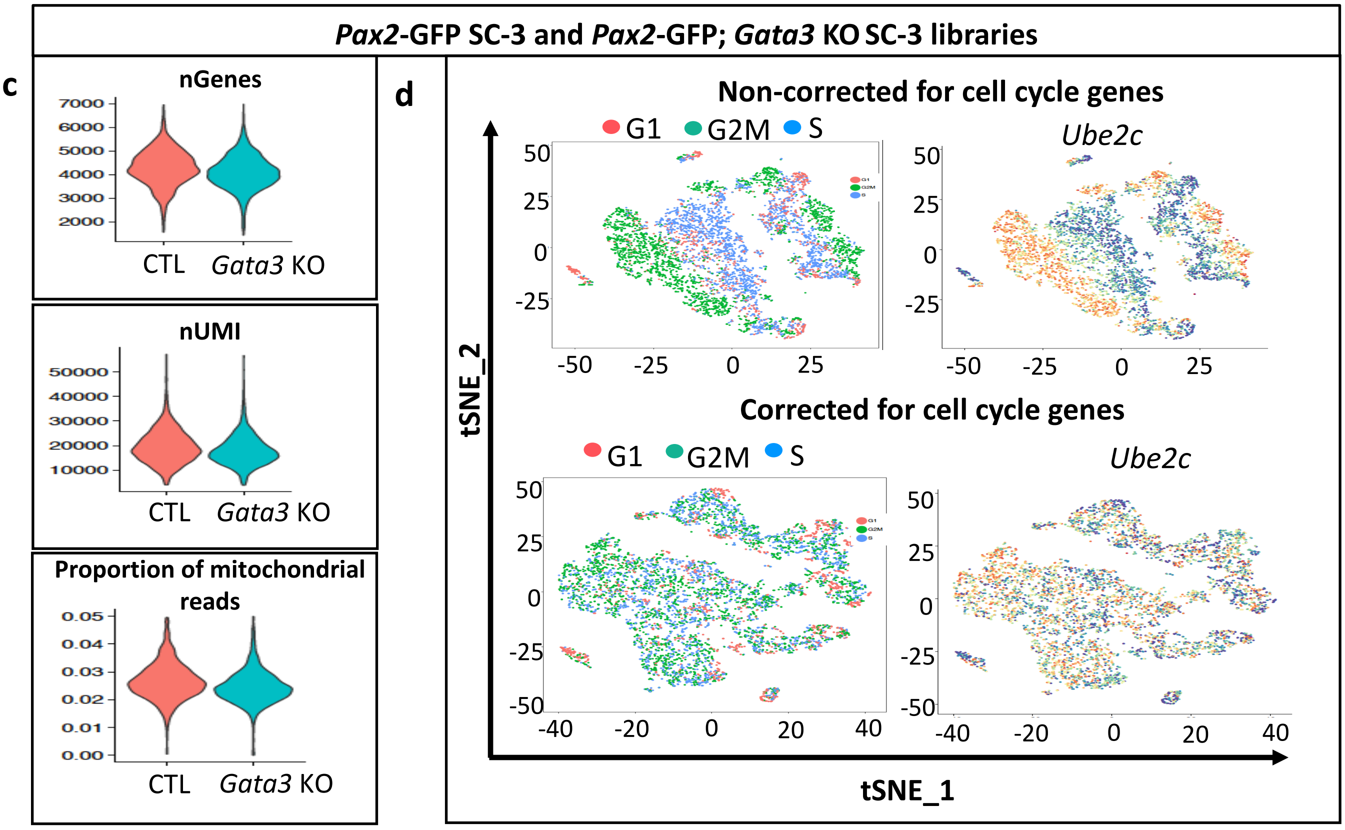

Supplementary Fig.1: Generation of single cell-RNA seq and Cluster-seq libraries from the trunk of mouse embryos by FACS. a Gating strategy used to sort *Pax2*-GFP+ single cells and cell clusters from the trunk of mouse embryos. Shown is the example for one of the libraries *Pax2*-GFP SC-2 and Cl-2. **b** Summary of the libraries used in the study. **c-d** Quality control and cell cycle bias removal. Shown is the example for CTL (*Pax2*-GFP-SC-3) and *Gata3* KO (*Pax2*-GFP;*Gata3* KO SC-3) libraries. **c** Comparison of number of genes, Unique Molecular Identifier (UMI) counts and percentage of mitosis. Source data are provided as a Source Data file. **d** tSNE analysis of merged libraries shows the correction of cell cycle bias. Upper panel (non-corrected); lower panel (corrected). Graphs on the left color code cells in function of the cell cycle stage (G1, G2M or S). Graphs on the right show expression of the cell cycle gene *Ube2c*.

Supplementary Fig.2

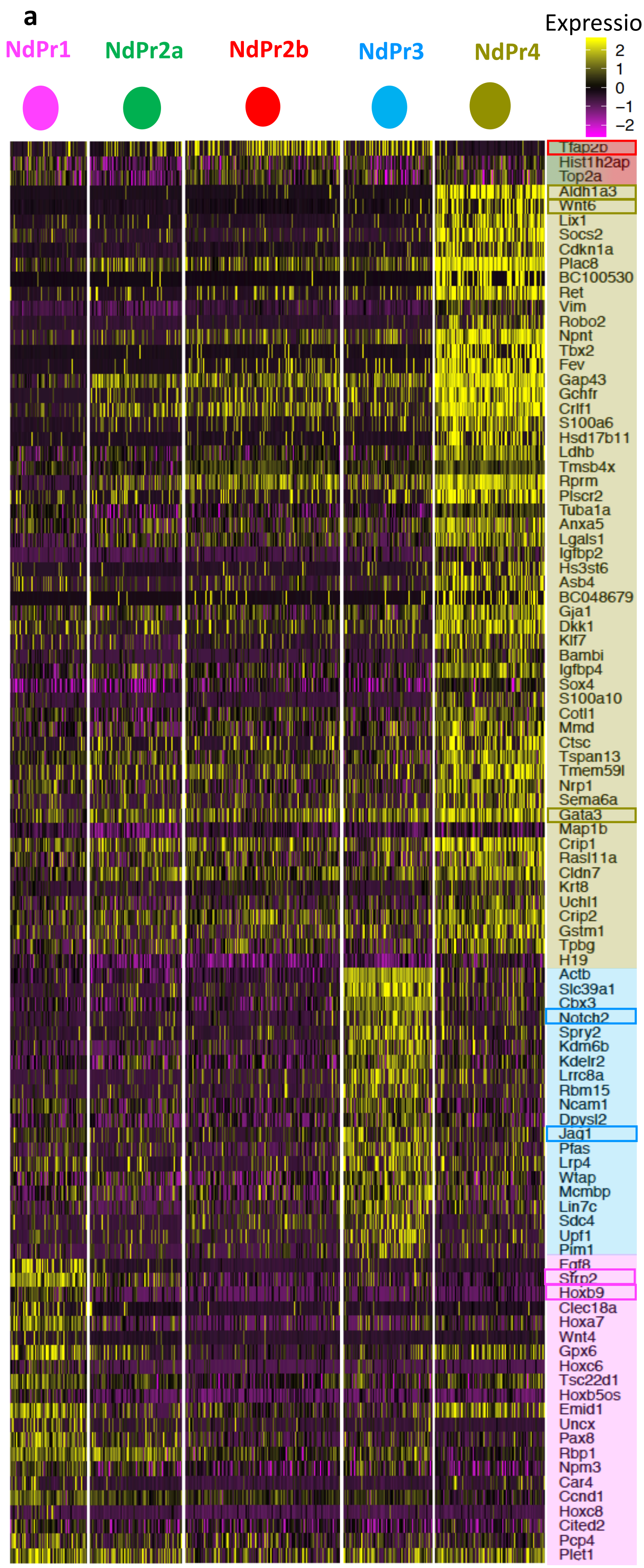

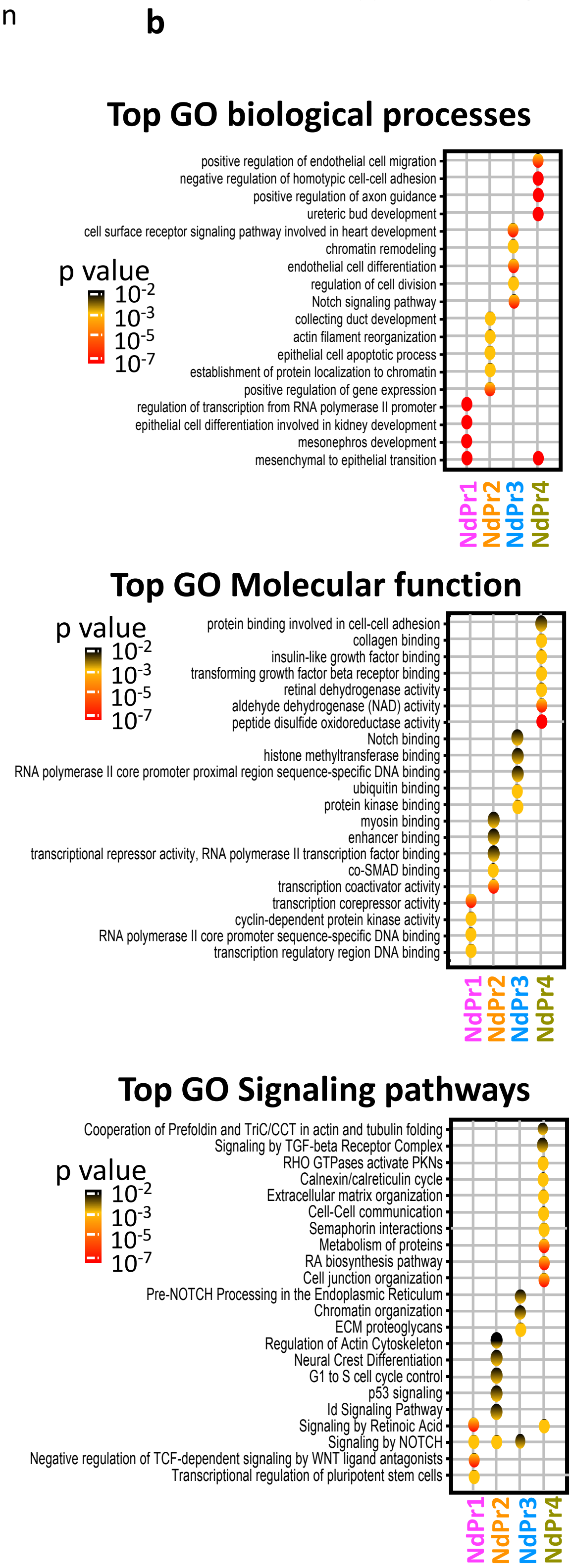

Supplementary Fig.2: Expression heatmap of most relevant NdPr markers and gene ontology of nephric duct cell populations. a Cell clusters are identified and color-coded. Cluster-defining genes were identified using a logFC > 0.25 and adjusted P_value < 0.05. Relevant cluster markers are denoted in a box. **b** Gene ontology analysis of nephric duct cell clusters.

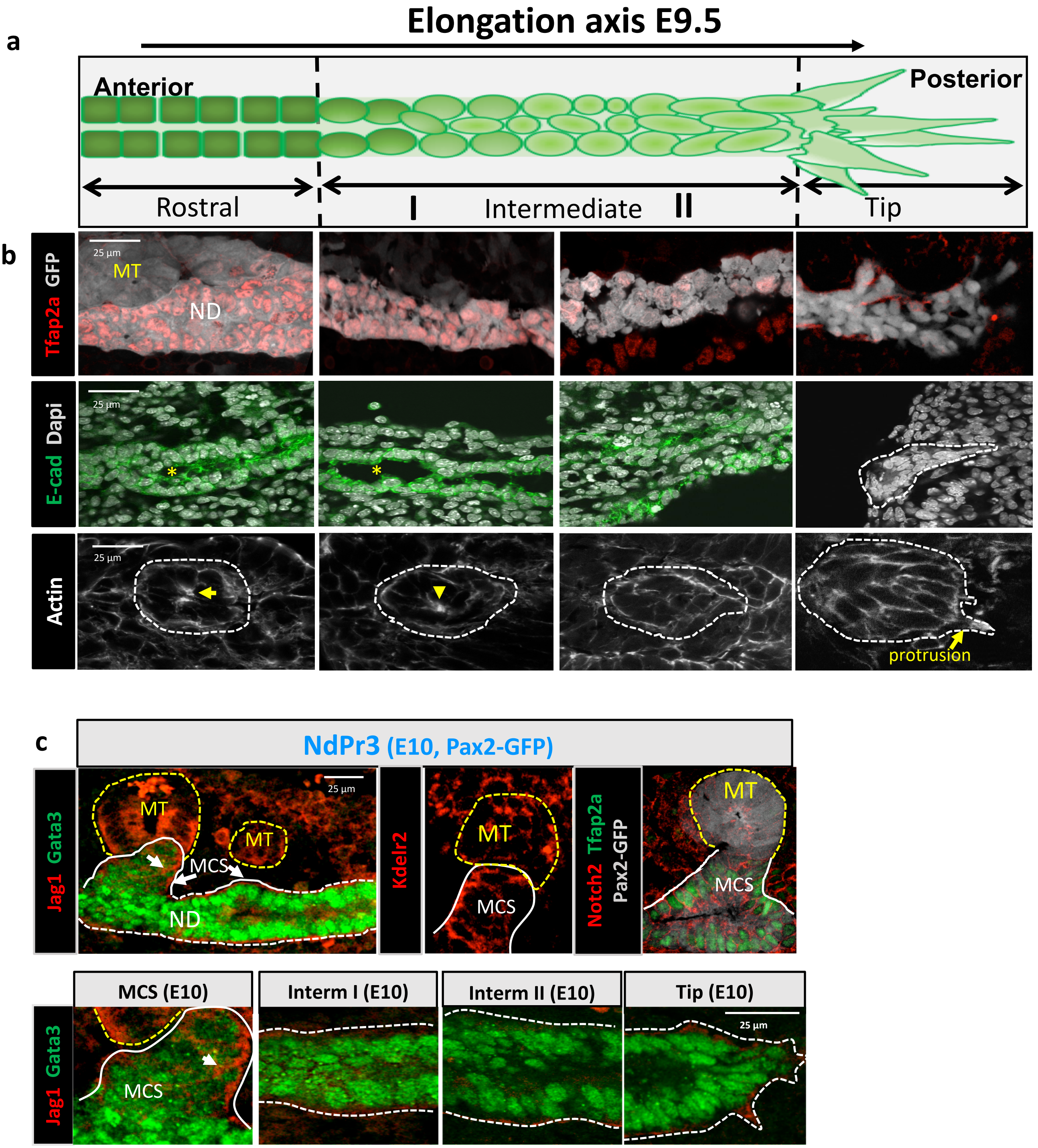

Supplementary Fig.3: Characterization of nephric duct progenitors cell populations. a Schematic representation of the elongating nephric duct at E9.5, depicting 4 regions (Rostral, Intermediate I, II and tip) according to actin dynamics, epithelialization and tubulogenesis profiles. **b** Immunostaining of Tfap2a and the epithelial marker E-cadherin in sagittal sections and F-actin (phalloidin) in transverse sections of *Pax2*-GFP nephric ducts at E9.5. Nephric duct lumen is denoted by a yellow asterisk, whereas the apical domain is marked by a yellow arrow. **c** Characterization of the NdPr3 population at E10.0. White and yellow dotted lines denote the nephric duct (ND) and mesonephric tubule (MT) regions respectively. White line denotes mesonephric connecting segment (MCS). Representative images of n=3 independent experiments. Scale bar 25 µm and the same for all the pictures on the same row.

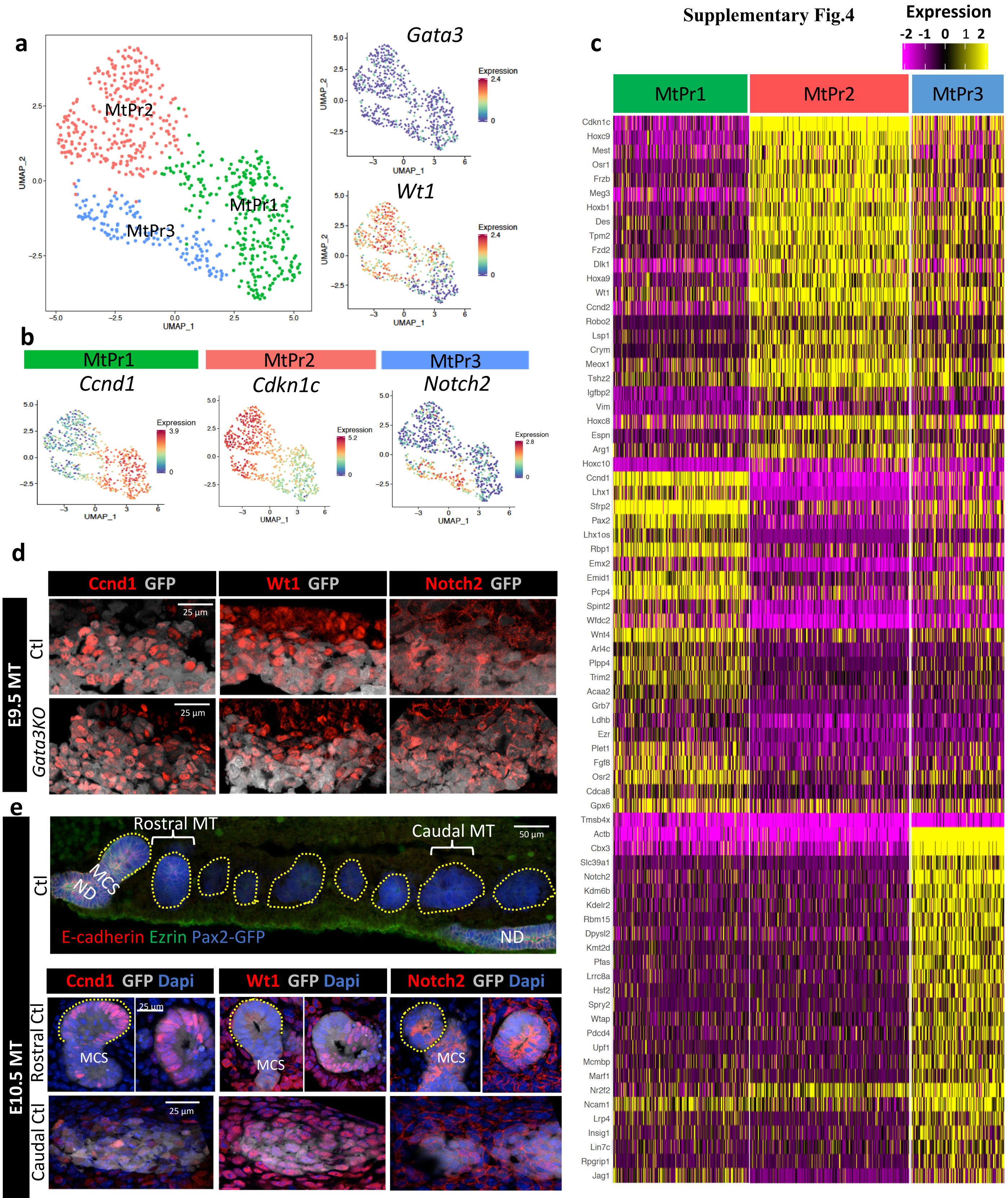

Supplementary Fig.4: Progenitor lineages in E9.5 mesonephric tubules. a Uniform Manifold Approximation and Projection (UMAP) analysis of the E9.5 mesonephric tubule lineage (*Wt1*+, *Gata3*-) identified 3 cell clusters (MtPr1, MtPr2 and MtPr3). **b** Expression of selected MtPr cluster markers in UMAP. **c** Expression heatmap of the top 25 markers per cluster (based on highest logFC). **d, e** Characterization of MtPr populations in *Pax2*-GFP and *Pax2*-GFP;*Gata3* KO embryos by immunofluorescence marker analysis in tissue sections at E9.5 (**d**) and E10.5 (**e**). Yellow dashed lines denote MT. ND: Nephric duct. MCS: mesonephric connecting segment. Representative images of n=3 embryos. Scale bar 25 µm and the same for all the pictures on the same row.

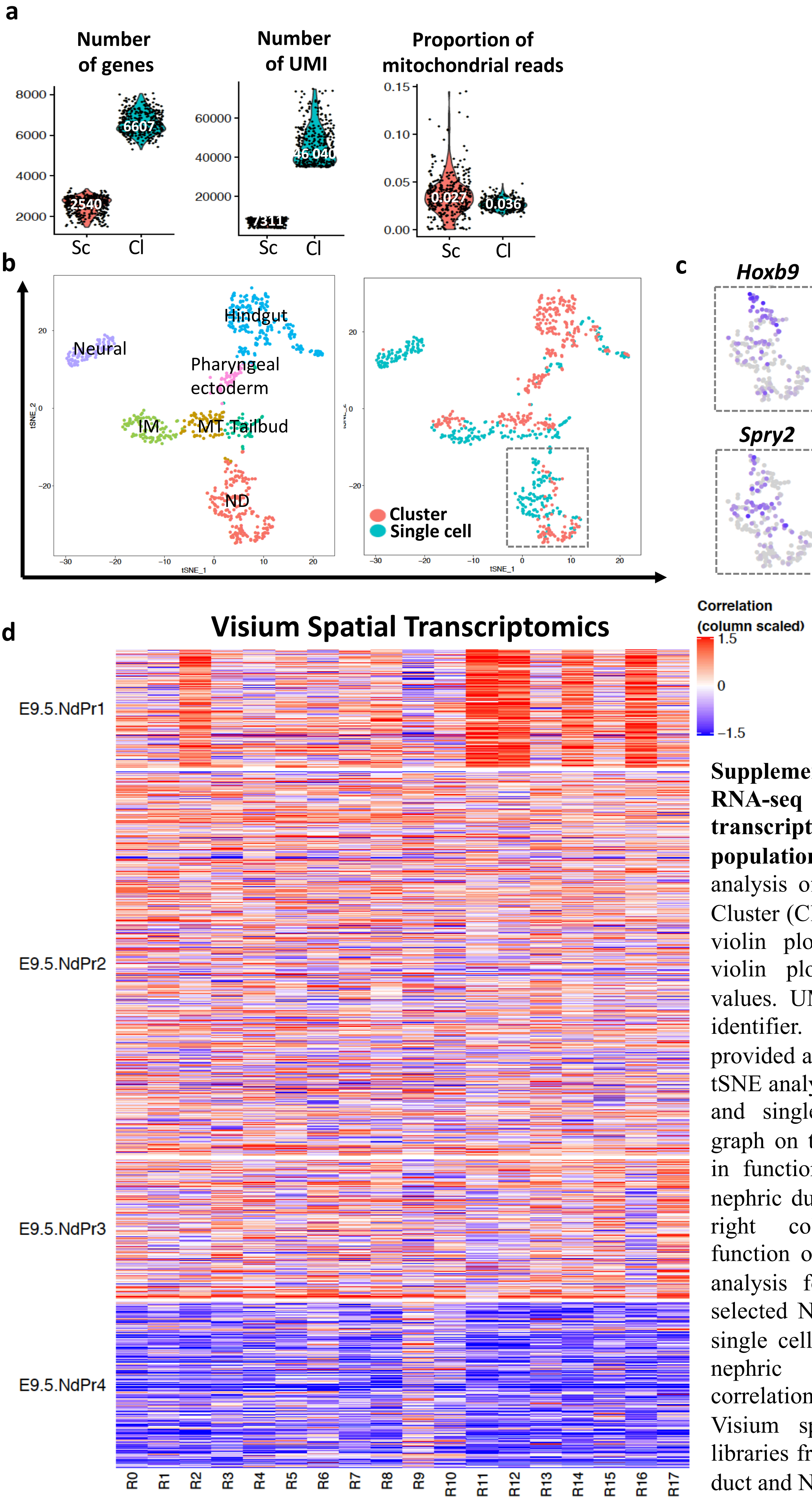

Supplementary Fig.5: Cluster-RNA-seq and spatial transcriptomics of NdPr populations. a Quality control analysis of single cell (Sc) and Cluster (Cl) libraries as shown is violin plots. Numbers on the violin plot denote the mean values. UMI: unique molecular identifier. Source data are provided as a Source Data file. **b** tSNE analysis of merged clusters and single cell libraries. The graph on the left is color-coded in function of cell types. ND: nephric duct. The graph on the right color-codes cells in function of the sample. **c** tSNE analysis for the expression of selected NdPr cluster marker in single cells and clusters for the nephric duct. **d** Pearson correlation heatmap between Visium spatial transcriptomics libraries from the rostral nephric duct and NdPr libraries at E9.5.

Supplementary Fig.6

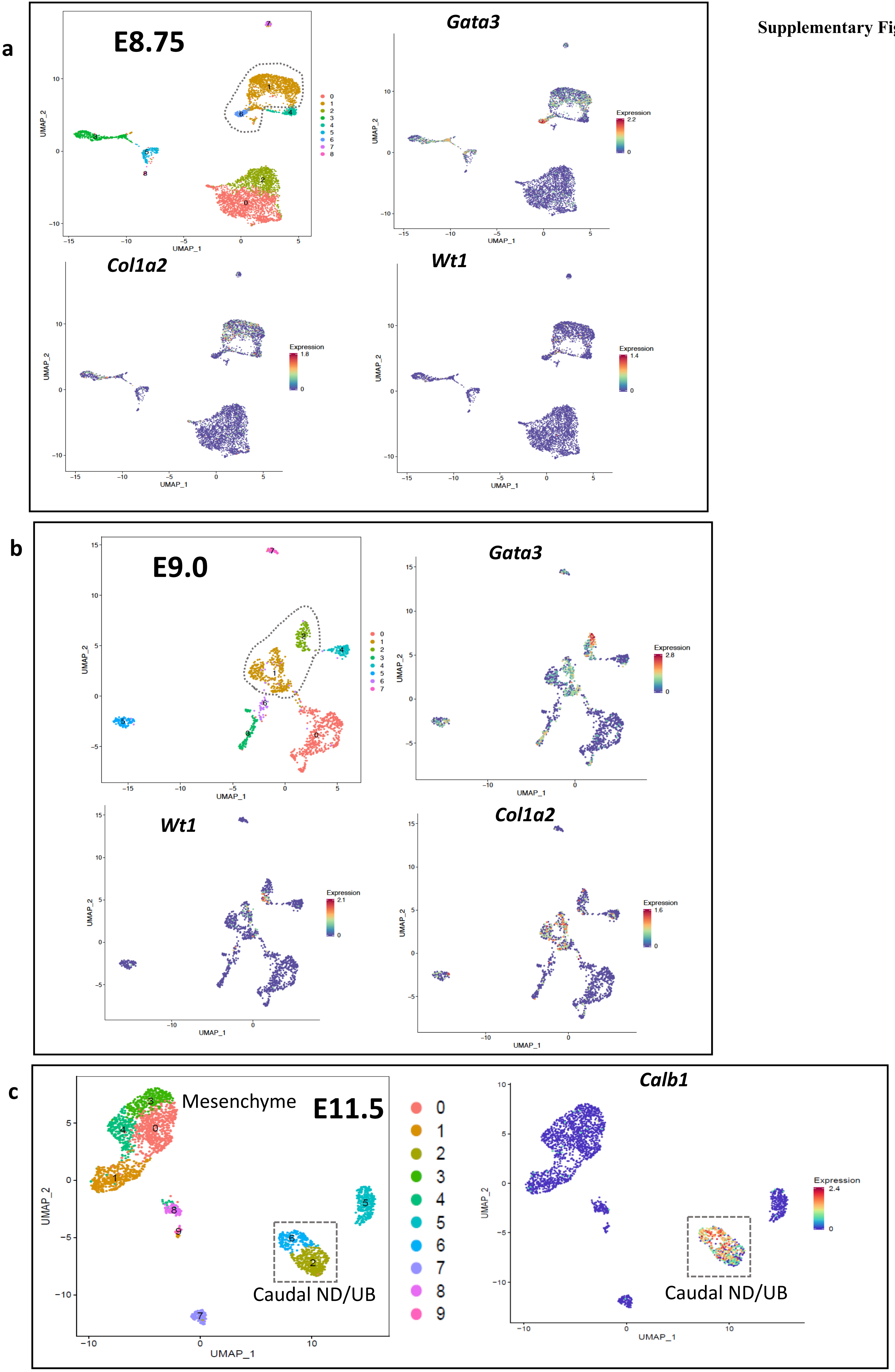

Supplementary Fig.6: Single cell RNA-seq of renal progenitors at different time points. Uniform Manifold Approximation and Projection (UMAP) analysis of *Pax2*-positive E8.75 (**a**) and E9.0 (**b**), as well as E11.5 urogenital system (**c**) single cell RNA-seq libraries. Relevant cell populations used in the study and including nephric duct (ND), intermediate mesoderm, mesonephric tubules and ureteric bud (UB) are denoted by dotted lines based on the expression of markers *Gata3*, *Col1a2*, *Wt1* and *Calb1*, respectively.

Supplementary Fig.7

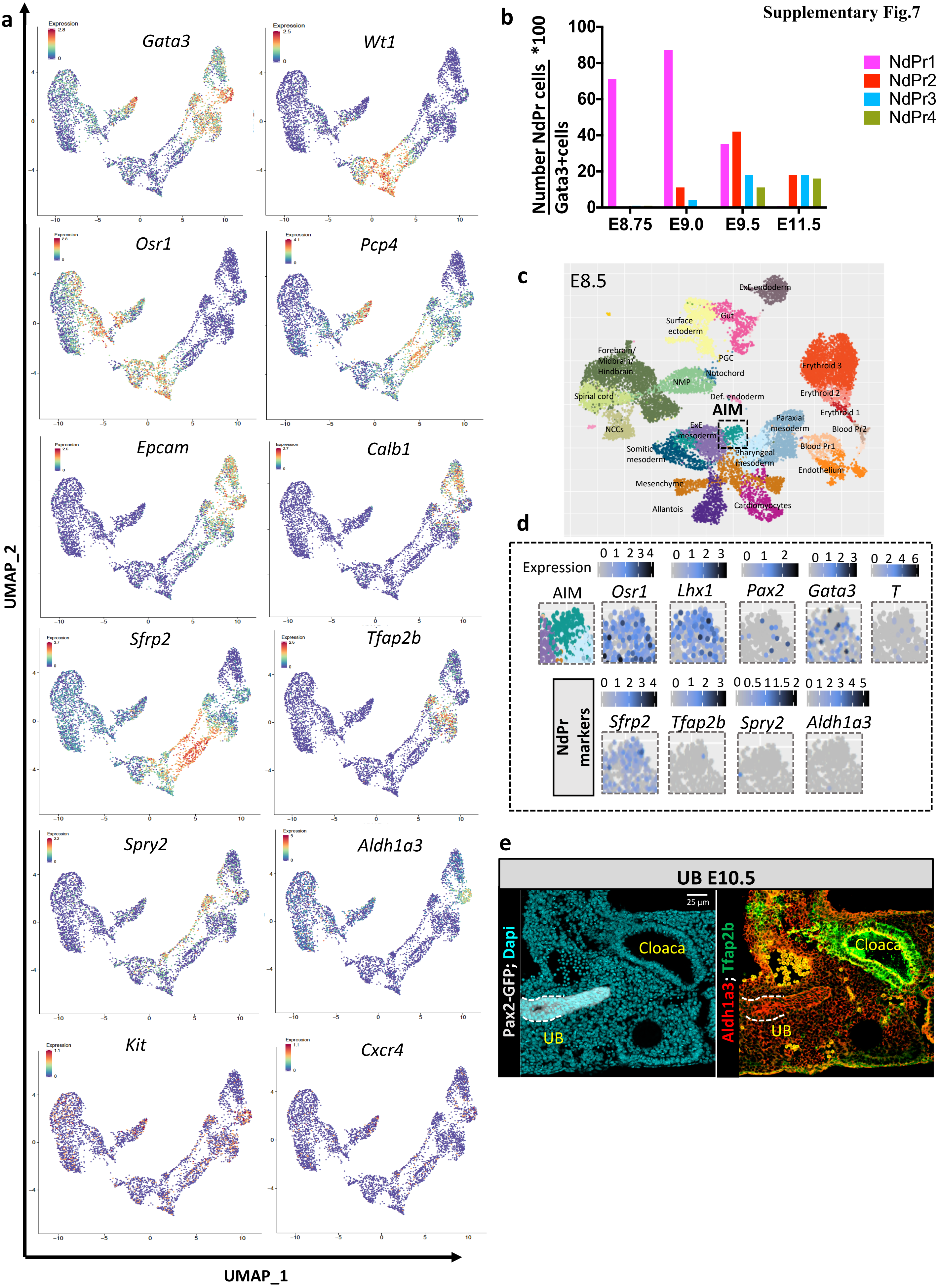

Supplementary Fig.7: Characterization of NdPr at different time points during early renal development. a Uniform Manifold Approximation and Projection (UMAP) analysis for the expression of selected cluster markers in intermediate mesoderm (IM), mesonephric tubules (MT), nephric duct (NdPr) and ureteric bud (UB) single cell RNA-seq libraries at E8.75, E9.0, E9.5 and E11.5. The UMAP depicting IM, MT, ND and UB clusters is shown in Figure 4A. **b** Percentage of each NdPr relative to the total number of ND or UB cells at four different time points (n=4 sc-RNA-seq libraries); n=1 library per time point. **c-d** Analysis of NdPr cluster markers in E8.5 anterior intermediate mesoderm (AIM) single cell RNA-seq data extracted from the Marioni lab's website atlas dataset. **e** Immunostaining for the NdPr4 and NdPr2 markers Aldh1a3 and Tfap2b in caudal ND tissue sections at E10.5 showing the presence of NdPr4 cell at the budding site. Nascent UB is depicted by white dotted lines. The angle of the section fails to show the connection between ND and cloaca that has already occurred at this stage. Representative image of n=3 embryos. Scale bar 25 μm and the same for all the pictures on the same row.

Supplementary Fig.8

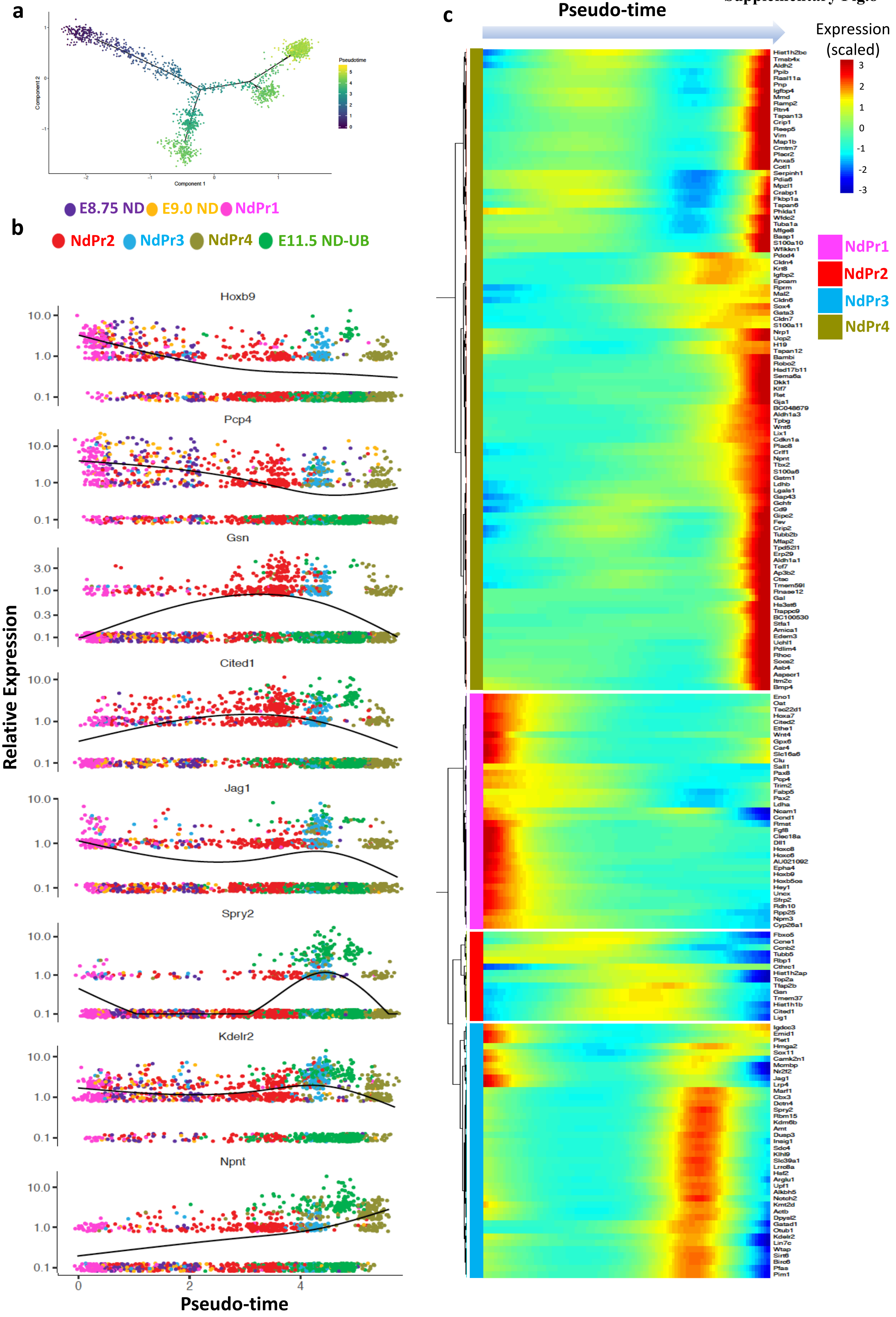

Supplementary Fig.8: Expression dynamics of NdPr cluster markers across pseudotime. a Principal component analysis showing the position of NdPr cell populations at E8.75, E9.0, E9.5 and E11.5 presented in Figure 4B, color coded by a pseudotime scale. **b** Representative expression profile of the selected NdPr cluster markers in pseudotime. Cells are color coded by NdPr type and time point. **c** Heatmap of the expression of NdPr cluster markers across pseudotime. Cell clusters are numbered and color-coded. Cluster-defining genes were identified using a logFC > 0.25 and adjusted P_value < 0.05. Genes are shown in rows. Cells (grouped in bins) are ordered according to pseudotime.

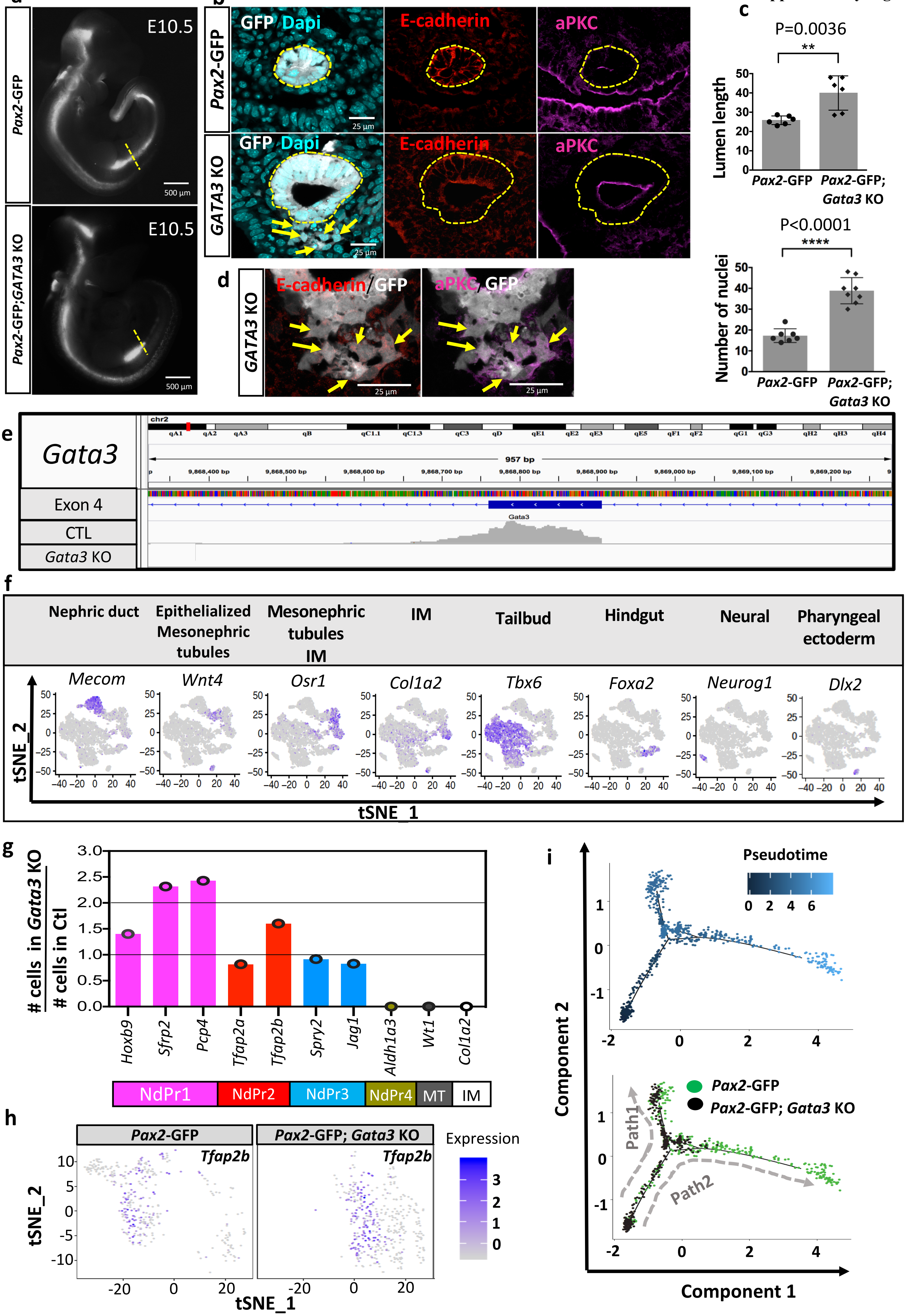
a
Pax2-GFP
E10.5
500 µm
Pax2-GFP;GATA3 KO
E10.5
500 µm
b
Pax2-GFP
GFP Dapi
E-cadherin
aPKC
25 µm
GATA3 KO
GFP Dapi
E-cadherin
aPKC
25 µm
c
P=0.0036
**
Lumen length
Pax2-GFP
Pax2-GFP; Gata3 KO
P<0.0001
****
Number of nuclei
Pax2-GFP
Pax2-GFP; Gata3 KO
d
GATA3 KO
E-cadherin/GFP
aPKC/GFP
25 µm
e
Gata3
Exon 4
CTL
Gata3 KO
chr2
957 bp
9,868,400 bp
9,868,500 bp
9,868,600 bp
9,868,700 bp
9,868,800 bp
9,868,900 bp
9,869,000 bp
9,869,100 bp
9,869,200 bp
f
Nephric duct
Epithelialized Mesonephric tubules
Mesonephric tubules IM
IM
Tailbud
Hindgut
Neural
Pharyngeal ectoderm
Mecom
Wnt4
Osr1
Col1a2
Tbx6
Foxa2
Neurog1
Dlx2
tSNE_2
tSNE_1
g
cells in Gata3 KO / # cells in Ctl
Hoxb9
Sfrp2
Pcp4
Tfap2a
Tfap2b
Spry2
Jag1
Aldh1a3
Wt1
Col1a2
NdPr1
NdPr2
NdPr3
NdPr4
MT
IM
h
Pax2-GFP
Pax2-GFP; Gata3 KO
Tfap2b
Expression
tSNE_2
tSNE_1
i
Pseudotime
Component 2
Component 1
Pax2-GFP
Pax2-GFP; Gata3 KO
Path1
Path2

Supplementary Fig.9: Characterization of the *Gata3* mutant nephric duct. **a** Wholemount GFP fluorescence in control (*Pax2*-GFP) and *Gata3* KO embryos (Pax2-GFP;*Gata3* KO) at E10.5. The yellow dotted line in the wholemount picture denote the region where the section was taken in (**b**). **b** Immunostaining for the polarity markers E-cadherin and aPKC in transversal cryosections. The yellow dotted line denotes the nephric duct whereas yellow arrows denote cells detaching from the nephric duct. Scale bar 25 μm and the same for all the pictures on the same row. **c** Quantification of lumen length and the number of nuclei per duct section, respectively. n=6 to 8 sections. Graphs represent mean ± SD and were compared by an unpaired t test. **d** Magnification view of the *Gata3* KO nephric duct (GFP signal) stained for E-cadherin and aPKC. Yellow arrows denote cells detaching from the nephric duct. Representative image of n=4 embryos. **e** Alignment of reads to Gata3 exon 4 in control and *Gata3* KO single-cell RNA-seq libraries. **f** tSNE analysis for the expression of markers of *Pax2* positive cell populations in the mouse embryo trunk (merged control and *Gata3* KO libraries). **g** Ratio of the number of cells expressing relevant markers in the *Gata3* mutant versus the control nephric duct library. **h** tSNE analysis of *Tfap2b* expression in control and *Gata3* KO nephric duct cells. **i** Principal component-based trajectory analysis of control and *Gata3* KO cells. Upper graph shows the superimposed trajectory color-coded by pseudotime whereas the lower graph color code by genotype: control (green cells) and *Gata3* KO (black cells).

Supplementary Fig.10

a

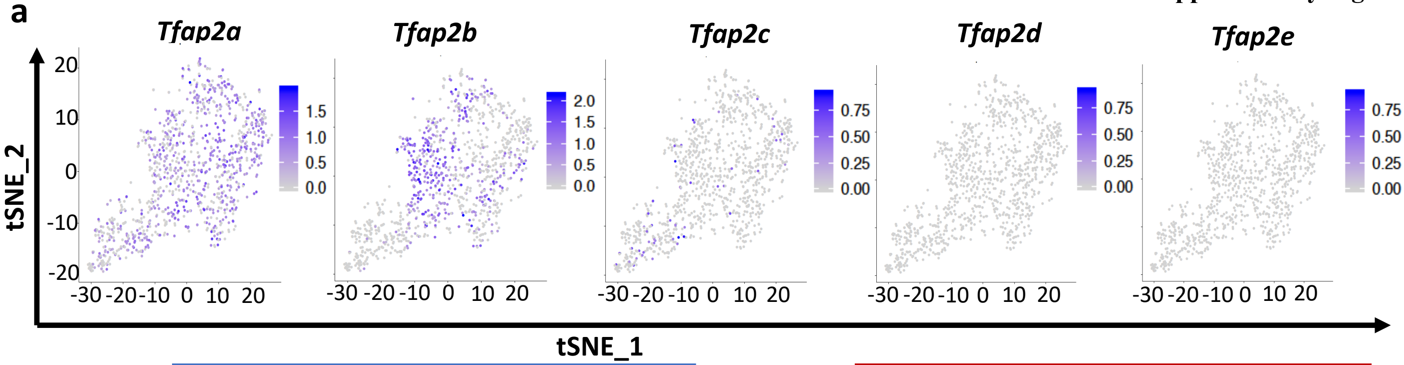

b

	Tfap2a Exon6		Tfap2b Exon4
sgRNA-1	5’-AAACTTTGATCCCAAGTCCG-3’	sgRNA-5	5’-GGCTTCATTAACACTACCGA-3’
sgRNA-2	5’-AGAAGCCGTCCACCTAGCCA-3’	sgRNA-6	5’-GGAGGAATGTCAGTCAACAC-3’
sgRNA-3	5’-TTCCGATCCCAATGAGCAAG-3’	sgRNA-7	5’-ATGCCTCAATGCATCTCTCC-3’
sgRNA-4	5’-AGTAAGGCATTTACCTCTGC-3’	sgRNA-8	5’-ACACAGGATTCTATTAGTGG-3’

c

	Tfap2a $^{+/-}$	Tfap2a $^{-/-}$	Tfap2b $^{+/-}$	Tfap2b $^{-/-}$	*Tfap2a$^{+/-}$;Tfap2b$^{-/-}$*	*Tfap2a$^{-/-}$;Tfap2b$^{+/-}$*	*Tfap2a$^{-/-}$;Tfap2b$^{-/-}$*
#/Total **(%)**	2/36 **(5.5)**	1/36 **(2.7)**	2/36 **(5.5)**	5/36 (13.8)	11/36 (30.5)	3/36 (8.3)	11/36 (30.5)
#/Total E9.5	1/9	0/9	1/9	2/9	0/9	0/9	4/9
#/Total E10.5	1/27	1/27	1/27	3/27	11/27	3/27	7/27
**Phenotype**	N/O	ONF	N/O	ND integrity defect	Severe ND integrity defect	ONF	ONF, ND elongation, Severe ND integrity, Ectopic cells 50% retarded E10.5

d

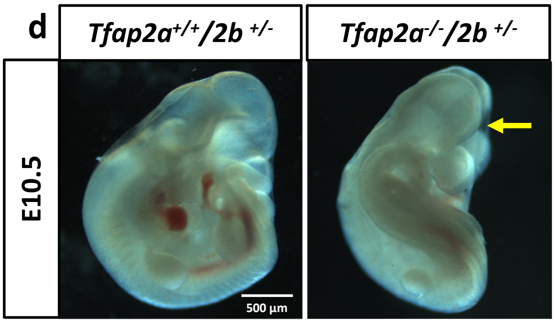

Supplementary Fig.10: Targeting of Tfap2a and Tfap2b by CRISPR/Cas9 technology. a tSNE analysis for the expression of *Tfap2* family members in the nephric duct. **b** Target sequence of the sgRNAs used to target *Tfap2a* and *Tfap2b*. **c** Summary of the genotypes, phenotypes and number of Tfap2a/2b mutants recovered. ONF: open neural fold, ND: nephric duct. **d** Wholemount transmitted light imaging of allelic series of *Tfap2a/2b* compound mutants shows open cranial neural fold (yellow arrow) when *Tfap2a* is knocked out. Scale bar 500 µm and the same for all the pictures.

Genotype	Primer name	Primer sequence
Pax2-GFP	Pax2-GFP_geno_Forward1	5’-ACTGGTGTGAGAGGCGGGTTCTT-3’
	Pax2-GFP_geno_Forward2	5’-GCTGGCGAAAGGGGGATGTGCTG-3’
	Pax2-GFP_geno_Reverse	5’-AAACAGGCAGAGTGGCTGAGTAT-3’
Gata3-GFP	Gata3-GFP_geno_Forward1	5’-GTCAGGGCACTAAGGGTTGTT-3’
	Gata3-GFP_geno_Forward2	5’-GGCCTACCCGCTTCCATTGCT-3’
	Gata3-GFP_geno_Reverse	5’-TATCAGCGGTTCATCTACAGC-3’
Tfap2a	Tfap2a_Exon6_geno_Forward	5’-TTTTCAAACCCTCTTGGCCTTGT-3’
	Tfap2a_Exon6_geno_Reverse	5’-GTCAACACAGAAAGAGACAGACT-3’
Tfap2b	Tfap2b_Exon4_geno_Forward	5’-TTCCCAGATGACTTGTGCTG-3’
	Tfap2b_Exon4_geno_Reverse	5’-AAGCAACTGCGTCAAACCTT-3’

Supplementary Table 1. Primer sequences used for genotyping mice or embryos by PCR.
