# Supplementary material for: A coordinated progression of progenitor cell states initiates urinary tract development
Source: Nat Commun. 2021 May 11;12:2627. doi: 10.1038/s41467-021-22931-5 (PMC8113267; doi:10.1038/s41467-021-22931-5)
Supplement: Supplementary file 3 — Reporting Summary [file 41467_2021_22931_MOESM3_ESM.pdf]

nature research

Corresponding author(s): Maxime Bouchard

Last updated by author(s): Feb 19, 2021

Reporting Summary

Nature Research wishes to improve the reproducibility of the work that we publish. This form provides structure for consistency and transparency in reporting. For further information on Nature Research policies, see our Editorial Policies and the Editorial Policy Checklist.

Statistics

For all statistical analyses, confirm that the following items are present in the figure legend, table legend, main text, or Methods section.

n/a	Confirmed	
[ ]	[x]	The exact sample size (*n*) for each experimental group/condition, given as a discrete number and unit of measurement
[ ]	[x]	A statement on whether measurements were taken from distinct samples or whether the same sample was measured repeatedly
[ ]	[x]	The statistical test(s) used AND whether they are one- or two-sided<br>*Only common tests should be described solely by name; describe more complex techniques in the Methods section.*
[x]	[ ]	A description of all covariates tested
[x]	[ ]	A description of any assumptions or corrections, such as tests of normality and adjustment for multiple comparisons
[ ]	[x]	A full description of the statistical parameters including central tendency (e.g. means) or other basic estimates (e.g. regression coefficient) AND variation (e.g. standard deviation) or associated estimates of uncertainty (e.g. confidence intervals)
[ ]	[x]	For null hypothesis testing, the test statistic (e.g. *F*, *t*, *r*) with confidence intervals, effect sizes, degrees of freedom and *P* value noted<br>*Give P values as exact values whenever suitable.*
[x]	[ ]	For Bayesian analysis, information on the choice of priors and Markov chain Monte Carlo settings
[x]	[ ]	For hierarchical and complex designs, identification of the appropriate level for tests and full reporting of outcomes
[ ]	[x]	Estimates of effect sizes (e.g. Cohen's *d*, Pearson's *r*), indicating how they were calculated

Our web collection on statistics for biologists contains articles on many of the points above.

Software and code

Policy information about availability of computer code

Data collection	Data were collected using GraphPad Prism software (version 6.0).
Data analysis	-FACS data was analyzed using FlowJo v10 (BD Biosciences) software.<br>-CRISPR/Cas9 targeting efficiency and analysis of indels was assessed with the web tools Tracking of Indels by Decomposition (TIDE), Inference of CRISPR Edits (ICE v2), Cas-Analyzer and Integrative Genomics Viewer (IGV) (version 2.5.3).<br>- For Single cell and Cluster RNA-seq, raw sequencing data for each sample was converted to matrices of expression counts using the Cell Ranger software (10X genomics, version 2.0.1).<br>-For Visium spatial transcriptomics, raw sequencing data for each sample was converted to matrices of expression counts using the Space Ranger software provided by 10X Genomics (version 1.0).<br>- Analysis of the Visium Spatial transcriptomics, the Single cell and Cluster RNA-seq data was performed using Seurat suite (versions 2.3.4 and 3.2.0). Pseudotime analyses were done with the R package Monocle 2 (version 2.8.0).<br>- Images were analyzed and processed using with Image J (Fiji) software version 2.0.0-rc-41 and NIS-Elements AR 5.11.00 (Nikon).<br>- Gene ontology enrichments analyses were performed using the Enrichr web-server.<br>- Statistical analyses were performed by using GraphPad Prism software (version 6.0).

For manuscripts utilizing custom algorithms or software that are central to the research but not yet described in published literature, software must be made available to editors and reviewers. We strongly encourage code deposition in a community repository (e.g. GitHub). See the Nature Research guidelines for submitting code & software for further information.

Data

Policy information about availability of data

All manuscripts must include a data availability statement. This statement should provide the following information, where applicable:

- Accession codes, unique identifiers, or web links for publicly available datasets
- A list of figures that have associated raw data
- A description of any restrictions on data availability

All Figures in the manuscript (except Figure6, Figure7, FigureS3 and FigureS10) have associated RNA sequencing raw data. All raw RNA sequencing data are publicly available in the Gene Expression Omnibus (GEO) data repository and can be downloaded via the following links:
https://www.ncbi.nlm.nih.gov/geo/query/acc.cgi?acc=GSE160136
https://www.ncbi.nlm.nih.gov/geo/query/acc.cgi?acc=GSE160137
https://www.ncbi.nlm.nih.gov/geo/query/acc.cgi?acc=GSE143806
Single cell RNA-seq data from E8.5 embryos was obtained from Marioni lab's website atlas dataset59 using the link https://marionilab.cruk.cam.ac.uk/MouseGastrulation2018/.
Source data are provided with this paper. Access to other data supporting the findings of this study will be granted upon request to the corresponding author.

Field-specific reporting

Please select the one below that is the best fit for your research. If you are not sure, read the appropriate sections before making your selection.

- [x] Life sciences
- [ ] Behavioural & social sciences
- [ ] Ecological, evolutionary & environmental sciences

For a reference copy of the document with all sections, see nature.com/documents/nr-reporting-summary-flat.pdf

Life sciences study design

All studies must disclose on these points even when the disclosure is negative.

Sample size	Sample size was not pre-determined using statistical methods. Experiments were performed with a sample size as large as possible and a minimum of n=3 independent replicates to perform statistical analyses and estimate the differences between means.
Data exclusions	No data were excluded from the analysis
Replication	Reproducibility of the experimental findings was verified by replicating the experiment minimum 3 times. All attempts at replication were successful.
Randomization	Samples were allocated into experimental groups based on the genotype or experimental conditions.
Blinding	For this study, the investigators were not blinded when allocating the different genotypes or treatments to avoid contamination between the strains or treatments.<br>However, for analysis of all data the investigators were blinded.

Reporting for specific materials, systems and methods

We require information from authors about some types of materials, experimental systems and methods used in many studies. Here, indicate whether each material, system or method listed is relevant to your study. If you are not sure if a list item applies to your research, read the appropriate section before selecting a response.

Materials & experimental systems

n/a	Involved in the study	
[ ]	[x]	Antibodies
[x]	[ ]	Eukaryotic cell lines
[x]	[ ]	Palaeontology and archaeology
[ ]	[x]	Animals and other organisms
[x]	[ ]	Human research participants
[x]	[ ]	Clinical data
[x]	[ ]	Dual use research of concern

Methods

n/a	Involved in the study	
[x]	[ ]	ChIP-seq
[ ]	[x]	Flow cytometry
[x]	[ ]	MRI-based neuroimaging

Antibodies

Antibodies used

Antibodies and dilutions used are as follows: E-cadherin (1:500, Invitrogen, ECCD-2); Hoxb9 (1:300, Santa Cruz, sc-398500); Tfap2b (1:100, Santa Cruz , sc-390119); Tfap2a (1:100, Santa Cruz, sc-12726); Jag1 (1:100, Santa Cruz, sc-390177); Notch2 (1:200, Cell Signaling, D76A6), Gata3 (1:100, Invitrogen, 14-9966-82); Aldh1a3 (1:100, Millipore Sigma, ABN427); Wt1 (1:300, Sigma-Aldrich, clone 6F-H2); Ccnd1 (1:100, Abcam, ab134175); Sfrp2 (1:100, Santa Cruz, sc-365524); Pcp4 (1:100, Atlas Antibodies, HPA005792); Kdelr2 (1:100, Santa Cruz, sc-57347), aPKCz/i (1:100, Santa Cruz, SC-216). Secondary antibodies and dilutions used are as follows: Alexa Fluor-555 or 647 secondary antibodies (1:500, Invitrogen); Alexa Fluor-635 conjugated phalloidin (1:40; Invitrogen).

Validation

1) E-cadherin monoclonal antibody (1:500, Invitrogen, ECCD-2).
Validation statement and information on the manufacturer web site:
https://www.thermofisher.com/antibody/product/E-cadherin-Antibody-clone-ECCD-2-Monoclonal/13-1900
Citations (190)
We previously published use of this antibody in doi: 10.1073/pnas.1707229114.

2) Hoxb9 (1:300, Santa Cruz, sc-398500).
Validation statement and information on the manufacturer web site:
https://www.scbt.com/p/hoxb9-antibody-h-8
Citations (5)

3) Tfap2b (1:100, Santa Cruz , sc-390119).
Validation statement and information on the manufacturer web site:
https://www.scbt.com/p/ap-2beta-antibody-c-6
Citations (2)

4) Tfap2a (1:100, Santa Cruz, sc-12726)
Validation statement and information on the manufacturer web site:
https://www.scbt.com/p/ap-2alpha-antibody-3b5
Citations (70)

5) Jag1 (1:100, Santa Cruz, sc-390177)
Validation statement and information on the manufacturer web site:
https://www.scbt.com/p/jagged1-antibody-e-12
Citations (12)

6) Notch2 (1:200, Cell Signaling, D76A6)
Validation statement and information on the manufacturer web site:
https://www.cellsignal.com/products/primary-antibodies/notch2-d76a6-xp-rabbit-mab/5732
Citations (55)

7) Gata3 (1:100, Invitrogen, 14-9966-82)
Validation statement and information on the manufacturer web site: https://www.thermofisher.com/antibody/product/Gata-3-Antibody-clone-TWAJ-Monoclonal/14-9966-82
Citations (31)

8) Aldh1a3 (1:100, Millipore Sigma, ABN427)
Validation statement and information on the manufacturer web site:
https://www.emdmillipore.com/CA/en/product/Anti-RalDH3-ALDH1A3,MM_NF-ABN427
Citations

9) Wt1 (1:300, Sigma-Aldrich, clone 6F-H2)
Validation statement and information on the manufacturer web site:
https://www.sigmaaldrich.com/catalog/product/mm/05753
Citations (21)

10) Ccnd1 (1:100, Abcam, ab134175)
Validation statement and information on the manufacturer web site:
https://www.abcam.com/cyclin-d1-antibody-epr2241-c-terminal-ab134175.html
Citations (497)

11) Sfrp2 (1:100, Santa Cruz, sc-365524)
Validation statement and information on the manufacturer web site:
https://www.scbt.com/p/frp-2-antibody-c-4
Citations (1)

12) Pcp4 (1:100, Atlas Antibodies, HPA005792)
Validation statement and information on the manufacturer web site:
https://www.atlasantibodies.com/products/antibodies/primary-antibodies/triple-a-polyclonals/pcp4-antibody-hpa005792/

Citations (17)

13) Kdelr2 (1:100, Santa Cruz, sc-57347)
Validation statement and information on the manufacturer web site:
https://www.scbt.com/p/kdel-receptor-antibody-kr-10

14) aPKCz/i (1:100, Santa Cruz, SC-216)
Validation statement and information on the manufacturer web site:
https://www.scbt.com/p/pkc-zeta-antibody-c-20
Citations (5)
We previously published use of this antibody in doi: 10.1016/j.stemcr.2017.02.004

Animals and other organisms

Policy information about studies involving animals; ARRIVE guidelines recommended for reporting animal research

Laboratory animals	Mouse housing conditions and procedures complied with the Canadian Council of Animal Care ethical guidelines and were approved by the McGill Animal Care Committee.<br>Mouse housing conditions:<br>-Mice were housed in autoclaved cages with free access to food and water, as well as appropriate and sufficient nesting and bedding material. Mice had a 12-hour cycle of light and darkness.<br>-Mouse rooms and cages were well ventilated and kept at a temperature range of 20-24 degrees Celsius, with a relative humidity of 45-65%.<br>For this study we used the following mouse strains and lines:<br>-C57Bl6 females reproductively active between 8-14 weeks of age for natural plugs or 3 weeks of age for superovulation and CRISPR/Cas9 microinjection.<br>-Pax2-GFP BAC transgenic males C57Bl6 background and reproductively active, between 2-8 months of age.<br>-Pax2-GFP;Gata3+/- transgenic males C57Bl6/C3H mixed background and reproductively active, between 2-8 months of age.<br>-Gata3+/- transgenic females, C57Bl6/C3H mixed background and reproductively active between 8-14 weeks of age for natural plugs.<br>-CD-1 pseudopregnant females (26-33 grams) between 8-14 weeks of age for CRISPR/Cas9 gene editing experiment.<br>-Pax2-GFP embryos (C57Bl6/C3H mixed background, sex: males and females) at embryonic day (E)8.75, 9.0, 9.5.<br>-Pax2-GFP;Gata3-/- embryos (C57Bl6/C3H mixed background, sex: males and females) at embryonic day (E)9.5.
Wild animals	The study did not involve wild animals
Field-collected samples	The study did not involve samples collected from the field
Ethics oversight	All animal experiments performed in this study were conducted in compliance with the Canadian Council of Animal Care ethical guidelines and were approved by the McGill Animal Care Committee.

Note that full information on the approval of the study protocol must also be provided in the manuscript.

Flow Cytometry

Plots

Confirm that:

- [x] The axis labels state the marker and fluorochrome used (e.g. CD4-FITC).
- [x] The axis scales are clearly visible. Include numbers along axes only for bottom left plot of group (a 'group' is an analysis of identical markers).
- [x] All plots are contour plots with outliers or pseudocolor plots.
- [x] A numerical value for number of cells or percentage (with statistics) is provided.

Methodology

Sample preparation	Mouse embryos were dissected in cold DMEM containing 10% fetal bovine serum (FBS) and 1% penicillin/streptomycin.<br>GFP positive single cells were isolated by FACS from dissociated trunk tissue. Material from both control and mutant was collected and processed at the same time. In brief, trunk tissue from stage-matched embryos (3-4 embryos per sample) was dissected and placed in 0.25% trypsin/EDTA at 37°C for 5 min, with pipette homogenization every 2 min.<br>The dissected UGS tissue was digested in 0.25% trypsin/EDTA at 37°C for 5 min, followed by Collagenase (1mg/mL) and Dispase (1 in 4 dilution) treatment in DMEM+ 10% FBS at 37°C for 30 min and finally 0.25% trypsin/EDTA at 37°C for 5 min. Dissociated cells were stained with an anti-Epcam antibody (Biolegend, 118233) and FACS sorted. A mixture of 50% Epcam +ve (epithelial cells including ND, UB and the cloaca epithelium), and 50% Epcam –ve (mesenchymal cells including the metanephric and surrounding caudal mesenchyme) was prepared for library preparation. For all samples, fixable Viability dye eFluor 780 staining was added to exclude dead cells. Cell clusters were isolated by sorting for higher unit size (FSC-A)

Instrument

Cells or clusters were sorted using a BD Biosciences FACSAria Fusion machine

Software

FACS data was analyzed using FlowJo v10 (BD Biosciences) software.

Cell population abundance

The abundance of post-sorted fractions for the each of the RNA-seq libraries is depicted in Supplementary Fig.1b and is as follows:
- SC-2: 13417 single cells
- Cl-2: 2658 cluster cells
- SC-3: 12178 single cells
- SC-3: 11774 single cells
- SC-4: 43147 single cells
- SC-5: 15760 single cells
- SC-6: 19895 single cells

Purity of the samples was assessed and confirmed by single cell RNA sequencing analysis.

Gating strategy

FSC/SSC was initially used to gate all cells/clusters excluding any debris. Then, the FSC/R780/60 was used to gate live cells. FSC-H/FSC-W was subsequently used to gate Single cells or Clusters (Not single cells). Finally, within the single/live cells population, B530/30-GFP/FSC-A was used to sort GFP/+ single cells (High GFP, low FSC) from E8.75, E9.0, E9.5 Pax2-GFP transgenic embryos or Epcam expressing renal cells from dissected E11.5 urogenital system. The same strategy B530/30-GFP/FSC-A was used to sort GFP/+ clusters from the cluster cell population (High GFP, but high FSC) of E9.5 Pax2-GFP embryos.
The gating strategy is also shown in details in Supplementary Fig.1a

☒ Tick this box to confirm that a figure exemplifying the gating strategy is provided in the Supplementary Information.
