# Supplementary material for: A coordinated progression of progenitor cell states initiates urinary tract development
Source: Nat Commun. 2021 May 11;12:2627. doi: 10.1038/s41467-021-22931-5 (PMC8113267; doi:10.1038/s41467-021-22931-5)
Supplement: Supplementary file 4 — Description of Additional Supplementary Files [file 41467_2021_22931_MOESM4_ESM.pdf]

Description of Additional Supplementary Files

File Name: Supplementary Data 1
Description: Mean Pearson correlation coefficients between NdPr single cell-, Cluster- and Visium spatial spot- RNA-seq samples. P-values reflect the statistical significance of the observed correlation, based on the random permutation of cluster cells.

File Name: Supplementary Data 2
Description: Differentially expressed genes in *Gata3* KO E9.5 nephric duct cells. The table shows the genes differentially expressed in *Gata3* KO (Pax2-GFP;Gata3KO-SC-3) versus control (Pax2-GFP SC-3) single cell RNA-seq libraries.

File Name: Supplementary Data 3
Description: Genotypes of Tfap2a/2b single and compound mutant embryos generated by CRISPR/Cas9 technology.
